# Road traffic noise is associated with increased cardiovascular morbidity and mortality and all-cause mortality in London

**DOI:** 10.1093/eurheartj/ehv216

**Published:** 2015-06-23

**Authors:** Jaana I. Halonen, Anna L. Hansell, John Gulliver, David Morley, Marta Blangiardo, Daniela Fecht, Mireille B. Toledano, Sean D. Beevers, Hugh Ross Anderson, Frank J. Kelly, Cathryn Tonne

**Affiliations:** 1 Department of Social and EnvironmentalHealth Research, London School of Hygiene and Tropical Medicine, Tavistock Place 15-17, London WC1H 9SH, UK; 2 UK Small Area Health Statistics Unit, MRC-PHE Centre for Environment and Health, Department of Epidemiology and Biostatistics, School of Public Health, Imperial College, London W2 1PG, UK; 3 Imperial College Healthcare NHS Trust, London, UK; 4 MRC-PHE Centre for Environment and Health, King's College London, Franklin-Wilkins Building, Waterloo SE1 9NH, UK

**Keywords:** Traffic noise, Cardiovascular, Mortality, Hospital admission, Epidemiology

## Abstract

**Aims:**

Road traffic noise has been associated with hypertension but evidence for the long-term effects on hospital admissions and mortality is limited. We examined the effects of long-term exposure to road traffic noise on hospital admissions and mortality in the general population.

**Methods and results:**

The study population consisted of 8.6 million inhabitants of London, one of Europe's largest cities. We assessed small-area-level associations of day- (7:00–22:59) and nighttime (23:00–06:59) road traffic noise with cardiovascular hospital admissions and all-cause and cardiovascular mortality in all adults (≥25 years) and elderly (≥75 years) through Poisson regression models. We adjusted models for age, sex, area-level socioeconomic deprivation, ethnicity, smoking, air pollution, and neighbourhood spatial structure. Median daytime exposure to road traffic noise was 55.6 dB. Daytime road traffic noise increased the risk of hospital admission for stroke with relative risk (RR) 1.05 [95% confidence interval (CI): 1.02–1.09] in adults, and 1.09 (95% CI: 1.04–1.14) in the elderly in areas >60 vs. <55 dB. Nighttime noise was associated with stroke admissions only among the elderly. Daytime noise was significantly associated with all-cause mortality in adults [RR 1.04 (95% CI: 1.00–1.07) in areas >60 vs. <55 dB]. Positive but non-significant associations were seen with mortality for cardiovascular and ischaemic heart disease, and stroke. Results were similar for the elderly.

**Conclusions:**

Long-term exposure to road traffic noise was associated with small increased risks of all-cause mortality and cardiovascular mortality and morbidity in the general population, particularly for stroke in the elderly.

**Clinical perspective**

Road traffic noise has been associated with hypertension but evidence for the long-term effects on hospital admissions and mortality is limited. Our results suggest small increased population risks of all-cause mortality and cardiovascular morbidity and mortality, particularly of stroke in the elderly, at moderate levels of road noise exposure. Findings are consistent with the larger body of evidence linking traffic noise exposure with hypertension.

## Introduction

The environmental burden of disease from traffic noise was recently estimated to be the second largest preceded only by airborne particulate matter.^1^ In one of Europe's largest cities, London, over 1.6 million people are exposed to daytime road traffic noise levels >55 dB^2^ which the WHO defines as a level of community noise that causes health problems,^3^ and a level that in the UK has been estimated to annually cause over 500 additional cases of hypertension-related myocardial infarctions and nearly 800 cases of stroke.^4^

Health effects of noise exposure are hypothesized to occur via several pathways.^5,6^ Exposure to noise may affect the autonomic nervous system increasing heart rate, blood pressure, and concentrations of noradrenaline, a stress hormone.^7^ Noise can also affect the hypothalamus–pituitary–adrenal axis leading to increased levels of cortisol, another stress hormone.^5^ In the long term, these reactions are suggested to promote low-grade inflammation and cardiovascular morbidity.^8^ Another suggested pathway is via sleep disorders^6,7^ some of which have been linked to an increased risk of mortality.^9^ Recently, long-term exposure to nighttime road traffic noise was linked to development of atherosclerosis,^10^ the pathology underlying a range of important cardiovascular diseases.

Epidemiological studies have reported associations between chronic exposure to environmental noise and health outcomes including annoyance, sleep problems, increase in blood pressure, and hypertension.^7,11–13^ Few studies, however, have specifically examined associations between road traffic noise and more severe health outcomes such as hospital admissions and mortality from cardiovascular diseases. Of these studies, most^14–19^ but not all^20,21^ have reported positive associations. Moreover, the independent long-term effects of road traffic noise and air pollutants remain to be confirmed,^22^ and there is lack of studies identifying susceptible population subgroups.^1,5^

Therefore, among 8.6 million Londoners, we aimed to quantify small-area effects of long-term exposure to road traffic noise, independent of air pollution, on all-cause and cardiovascular mortality as well as on cardiovascular hospital admissions in adult and elderly populations. We hypothesized that higher noise levels are associated with greater risk of morbidity and mortality. We also examined possible effect modification of these associations by area-level socioeconomic deprivation.

## Methods

### Study area and population

**Figure 1:**
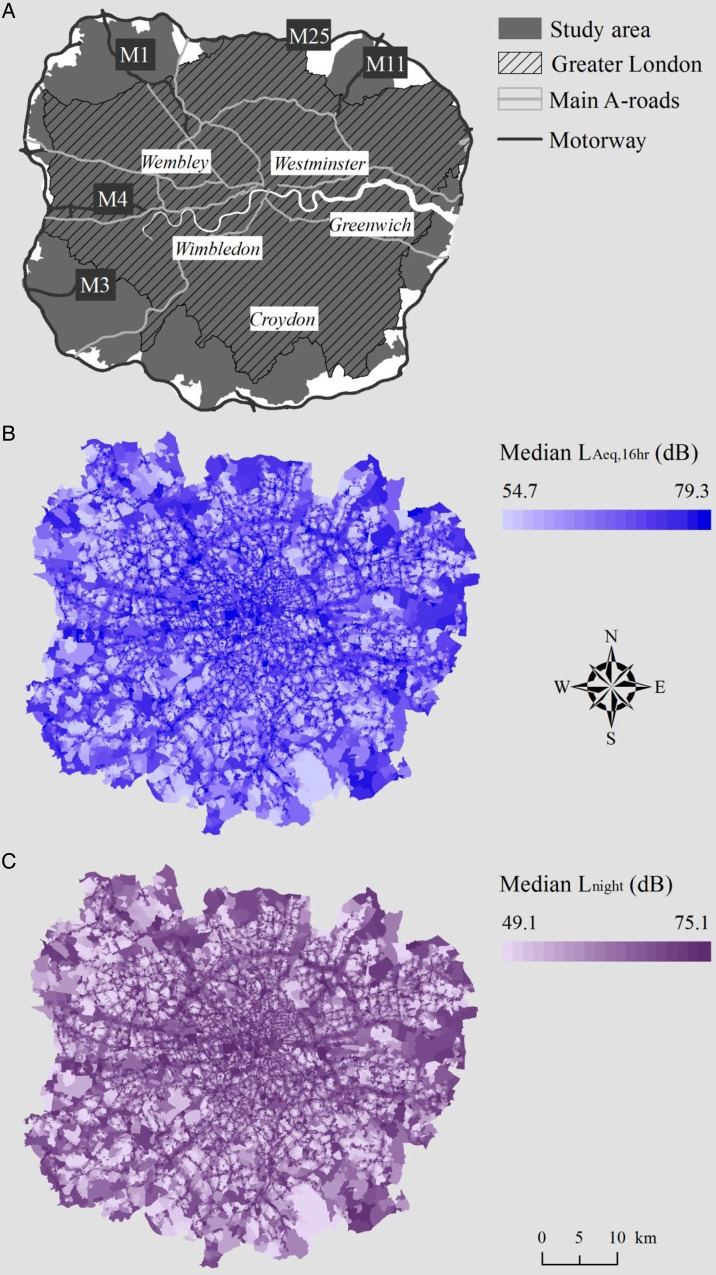
Map of the study area (*A*), and distribution of median day- (*B*) and nighttime (*C*) road traffic noise at census output area level.

The study area was that inside the M25 motorway which includes Greater London (*Figure 1*); hereafter referred to as London. We used census output areas (COAs) with a mean population of 300 (>40 households)^23^ for the hospital admission analyses, and census geographical unit Lower Layer Super Output Areas (LSOA) with a mean population of 1500 (>400 households)^24^ for the mortality analyses. We included 27 686 COAs and 5358 LSOAs with complete information for noise exposure, outcomes, and possible area-level confounders in the analyses.

### Outcomes

We used the first registered emergency hospital episode of each year (international classification of diseases (ICD) 10th revision codes) for all cardiovascular causes (I00–I99), ischaemic heart disease (IHD, I20–I25), and stroke (I61, I63, I64). Deaths were classified according to the underlying cause on the death certificate. We used deaths from all natural causes (A00–R99), all cardiovascular causes (I00–I99), IHD (I20–I25), and stroke (I61, I63, I64). These data also included the person's age, sex, and postcode of residential address at the time of admission or death. To ensure sufficient numbers for the analyses, we aggregated annual admission counts to COAs and death counts to LSOAs by sex and 5-year age bands. We then used annual mid-year population estimates by sex and 5-year age bands to calculate standardized mortality and admission rates for each area. Hospital admission data were derived from Hospital Episode Statistics and are held by the UK Small Area Health Statistics Unit (SAHSU). The mortality and population data were supplied by the Office for National Statistics, derived from the national mortality registrations and the Census, and are held by SAHSU. Data use was covered by approvals from the National Research Ethics Service—reference 12/LO/0566 and 12/LO/0567—and National Information Governance Board and Ethics and Confidentiality Committee approval for section 251 support (NIGB—ECC 2-06(a)/2009). The study was conducted in accordance with the Helsinki declaration.

### Traffic noise exposure

Annual road traffic noise levels for the years 2003–10 were modelled at geometric centroids of ∼190 000 postcode locations using the TRAffic Noise EXposure (TRANEX)^2^ model with 0.1 dB(A) noise-level resolution. As the geometric centroid of the postcode usually falls on a building, we universally moved them to 1 m from the facade of the nearest residential dwelling. This model uses detailed information on traffic for ∼63 000 road links including varying flows and speeds for each year in the study period, and detailed information on land cover and heights of individual buildings. Validation studies conducted in two UK cities showed Spearman's correlation between modelled and measured noise levels to be high: 0.90 (*P*-value < 0.0001), suggesting good model performance. Exposures to day- and nighttime noise were estimated, and health effects examined, separately as suggested by WHO.^12^ For daytime exposure, we used L_Aeq,16hr_ which is the average of 16 hourly measures from 7:00 to 22:59. For nighttime, we used L_night_ that averages hourly measures from 23:00 to 6:59. For the hospital admission analyses noise data were aggregated to COAs by taking the median across all annual postcode address centroid noise levels within a COA, and median of these annual medians COA-level values over 2003–10. Similarly, we calculated LSOA-level noise estimates for the mortality analyses. Noise estimates were categorized by 5 dB increments—<55 (reference), 55–60, and >60 dB—as well as by tertiles.

### Confounders

We obtained area-level data on Carstairs deprivation index (based on unemployment, overcrowding, car ownership, and low social class), and percentage of black and South Asian ethnicity from the UK Census 2011 provided by the Office for National Statistics. We used annual smoothed and age and sex standardized relative risks (RRs) for lung cancer mortality (ICD-10: C33-C34) at the LSOA and COA level as a proxy for area-level smoking.^25^ Air pollution is another possible confounder of the association between noise and health,^22^ thus we used median fine particle (particulate matter with diameter <2.5 µm, PM_2·5_) and nitrogen oxide (NO*_x_*, for sensitivity analysis) concentrations averaged over 2003–10 both aggregated to LSOA and COA levels. These exposure estimates were obtained for the whole study area using KCLurban dispersion modelling system which incorporates hourly meteorological measurements, empirically derived NO–NO_2_–O_3_ and PM relationships, and information on source emissions from the London Atmospheric Emissions Inventory.^26^

### Statistical methods

To model spatial dependencies between nearby areas, we used ecological Poisson regression models speciﬁed in a Bayesian framework that can be implemented through the Integrated Nested Laplace Approximation (INLA) approach.^27^ We calculated age and sex standardized expected number of deaths and hospital admissions for each small area and included these as offsets in the models. Following the Besag-York-Mollie specification, we modelled the spatial dependence (spatially structured residual) through an intrinsic conditional autoregressive structure, and non-spatial heterogeneity (unstructured residual), which is due to the unobserved non-spatial variables, through a random effect.

The regression models were adjusted for quintiles of socioeconomic deprivation; tertiles of both black and South Asian ethnicities as well as continuous smoking indicator, and fine particle concentration. We performed all analyses for the adult (≥25 years) and the elderly (≥75 years) populations, as old people may be more vulnerable to the effects of noise than young.^3^ People living in the deprived vs. affluent areas often have higher risk of mortality and morbidity,^28^ we therefore tested for possible effect modification by area-level deprivation using an interaction term (noise × deprivation). As sensitivity analyses, we ran models without adjustment for air pollution and adjusting the models for NO*_x_* instead of PM_2.5_. All analyses were run with R 3.1.0^29^ using the package R-INLA (www.r-inla.org). The results are presented as RRs with 95% credible intervals (CIs) with the lowest exposure group as the reference in categorical analyses, and per 5 dB increase in linear models.

## Results

In the study population of 8.61 million, the total number of hospital admissions from cardiovascular causes was 400 494 among adults, and 179 163 among the elderly between 2003 and 2010. There were a total of 442 560 and 291 139 deaths among adults and the elderly, respectively. Distributions of cases across the small areas by age groups are presented in *Table 1*.

**Table 1:** Summary statistics of cardiovascular hospital admissions and all-cause and cardiovascular mortality within small areas in London (2003–10)

Outcome (ICD 10-codes^a^)	Total *N*	Mean	SD	Median	Min	Max
Hospital admissions		COA				
All cardiovascular (I00–I99)						
Adults (≥25 years)	400 494	15	9	13	0	146
Elderly (≥75 years)	179 163	6.5	6	5	0	135
Ischaemic heart disease (I20–I25)						
Adults	133 688	4.8	4	4	0	45
Elderly	48 544	1.8	2	1	0	32
Stroke (I61, I63, I64)						
Adults	62 513	2.3	2	2	0	37
Elderly	35 697	1.3	2	1	0	30
Mortality		LSOA				
All-cause (A00–R99)						
Adults (≥25 years)	442 560	83	48	71	0	483
Elderly (≥75 years)	291 139	54	43	43	0	444
All cardiovascular (I00–I99)						
Adults	151 585	28	18	25	0	221
Elderly	108 269	20	16	16	0	212
Ischaemic heart disease (I20–I25)						
Adults	69 163	13	7	12	0	105
Elderly	45 968	9	6	7	0	99
Stroke (I61, I63, I64)						
Adults	29 036	5	5	4	0	61
Elderly	23 245	4	5	3	0	56

COA, census output area; LSOA, lower layer super output area.

^a^ICD 10th version.

Distributions of day- and nighttime noise levels across COAs and LSOAs are presented in *Table 2*. Median daytime exposure at both COA and LSOA level was 55.6 dB, for nighttime exposure, these figures were 50.2 and 50.1 dB, respectively. A map of the study area and the distribution of day- and nighttime noise levels are shown in *Figure 1*. Little variation was observed in noise levels by quintiles of area-level deprivation or tertiles of ethnicity (Supplementary material online, *Table S1*). Correlations between linear day- and nighttime road noise estimates were 0.99 at both COA and LSOA levels. However, the distributions of day- and nighttime noise were different; for example, only 133 of the 16 174 COAs belonging to the daytime 55–60 dB category had the same level of nighttime exposure. Correlations between road noise and PM_2.5_ ranged from 0.39 to 0.45, and those between noise and NO*_x_* from 0.42 to 0.48 (Supplementary material online, *Table S2*).

**Table 2:** Summary statistics of median road traffic noise levels (dB) and air pollution concentrations (µg/m^3^) across small areas in London (2003–10)

Exposure	Mean	SD	10th %	33th %	Median	66th %	90th %
COA (*n* = 27 686)							
L_Aeq,16 h_	57.7	4.19	54.9	55.2	55.6	56.9	64.6
L_night_	52.4	4.45	49.3	49.7	50.2	51.7	59.7
PM_2.5_	15.1	0.85	14.1	14.6	15.0	15.4	16.2
NO*_x_*	65.7	15.8	46.6	57.3	63.8	70.7	86.5
LSOA (*n* = 5358)							
L_Aeq,16 h_	56.6	2.54	54.9	55.2	55.6	56.2	59.6
L_night_	51.2	2.76	49.3	49.7	50.1	50.8	54.7
PM_2.5_	15.0	0.80	14.1	14.6	15.0	15.3	16.1
NO*_x_*	64.4	14.5	46.2	56.8	63.1	69.1	83.7

COA, census output area; LSOA, lower layer super output area.

### Hospital admissions

Daytime road traffic noise was significantly associated with hospital admissions for stroke in adults with RR 1.05 (95% CI 1.02–1.09) in areas exposed to >60 vs. <55 dB. There were no statistically significant association for the other outcomes (*Table 3*) Among the elderly, daytime noise was associated with all cardiovascular disease and stroke admissions; RRs were highest for stroke: 1.09 (95% CI 1.04–1.14) in areas >60 vs. <55 dB. Nighttime noise was also associated with increased risk of stroke admission, but not with the other admission outcomes (*Table 3*). Results using noise tertiles were similar although the effect estimates between daytime noise and stroke attenuated slightly, and those between nighttime noise and stroke became stronger (Supplementary material online, *Table S3*). Not adjusting for air pollution attenuated the effect estimates for stroke slightly, but adjusting for NO*_x_* did not change the results (Supplementary material online, *Table S4*).

**Table 3:** Adjusted^a^ relative risks for cardiovascular hospital admissions by categorized day- and nighttime road traffic noise

Noise dB	*n* of COAs	All cardiovascular diseases				Ischaemic heart disease				Stroke			
		Mean *n*^b^	RR	95% CI		Mean *n*^b^	RR	95% CI		Mean *n*^b^	RR	95% CI	
Adults													
L_Aeq, 16 h_													
<55	5735	15	1			5	1			2	1		
55–60	16 174	15	1.01	1.00	1.02	5	1.00	0.98	1.02	2	1.04	1.02	1.07
>60	5777	13	1.00	0.99	1.02	4	1.00	0.97	1.02	2	1.05	1.02	1.09
L_night_													
<55	21 988	15	1			5	1			2	1		
55–60	3098	14	0.99	0.97	1.00	5	0.99	0.97	1.01	2	1.02	0.99	1.05
>60	2600	12	1.00	0.99	1.02	4	1.00	0.98	1.04	2	1.01	0.98	1.05
Elderly													
L_Aeq, 16 h_													
<55	5735	7	1			2	1			1	1		
55–60	16 174	7	1.02	1.01	1.04	2	1.01	0.98	1.04	1	1.06	1.03	1.09
>60	5777	6	1.02	0.99	1.04	2	1.01	0.97	1.05	1	1.09	1.04	1.14
L_night_													
<55	21 988	7	1			2	1			1	1		
55–60	3098	6	0.99	0.97	1.01	2	0.99	0.96	1.03	1	1.05	1.01	1.09
>60	2600	5	1.00	0.97	1.02	1	1.00	0.97	1.06	1	1.02	0.97	1.08

COA, census output area.

^a^Adjusted for age, sex, area-level deprivation, ethnicity, smoking, and PM_2.5_.

^b^Mean number of hospital admissions per COA.

In the linear models, we observed positive non-significant associations for day- and nighttime noise with stroke admissions among adults and the elderly, but for the other outcomes associations were weaker (Supplementary material online, *Table S5*). Interactions for day- and nighttime noise with area-level deprivation were non-significant in all admission models.

### Mortality

Daytime noise was associated with small but statistically significant increases in all-cause mortality in adults; the RR was 1.04 (95% CI 1.00–1.07) in areas >60 vs. <55 dB(A) (*Table 4*). Associations were mainly positive but non-significant for daytime noise and cardiovascular, IHD, and stroke mortality. Among the elderly, associations were similar to those in adults (*Table 4*) but there was a larger non-significant association with stroke mortality with RR of 1.06 (95% CI 0.97–1.15) in areas >60 vs. <55 dB. Associations for nighttime noise in both age groups were close to null except for stroke mortality (RR 1.13, 95% CI 0.98–1.30 among elderly). Results using tertiles of noise were of similar magnitude, but associations between daytime noise and cardiovascular mortality in both age groups, and nighttime noise and stroke in the elderly reached statistical significance, possibly due to greater power (i.e. higher numbers) in the highest exposure group (Supplementary material online, *Table S6*). Not adjusting for air pollution attenuated the effect estimates slightly but adjustment for NO*_x_* rather than PM_2.5_ had only minor effects on the results (Supplementary material online, *Table S7*).

**Table 4:** Adjusted^a^ relative risks for all-cause and cardiovascular mortality by categorized day- and nighttime road traffic noise

Noise dB	*n* of LSOAs	All-causes				All cardiovascular				Ischaemic heart disease				Stroke			
		Mean *n*^b^	RR	95% CI		Mean *n*^b^	RR	95% CI		Mean *n*^b^	RR	95% CI		Mean *n*^b^	RR	95% CI	
Adults																	
L_Aeq, 16 h_																	
<55	873	82	1			29	1			13	1			6	1		
55–60	3994	84	1.03	1.01	1.05	29	1.02	0.99	1.05	13	1.03	1.00	1.06	5	1.00	0.96	1.05
>60	491	73	1.04	1.00	1.07	24	1.03	0.98	1.07	11	1.03	0.99	1.08	4	1.01	0.93	1.09
L_night_																	
<55	4866	84	1			29	1			13	1			6	1		
55–60	362	74	1.00	0.97	1.03	25	1.01	0.97	1.04	11	1.01	0.97	1.05	4	0.98	0.91	1.05
>60	130	64	1.00	0.95	1.05	19	0.96	0.90	1.02	8	0.93	0.86	1.00	4	1.08	0.95	1.22
Elderly																	
L_Aeq, 16 h_																	
<55	873	54	1			21	1			9	1			5	1		
55–60	3995	55	1.03	1.01	1.05	20	1.02	1.00	1.05	9	1.04	1.01	1.07	4	1.02	0.97	1.07
>60	491	47	1.04	1.00	1.08	17	1.04	0.99	1.09	7	1.05	0.99	1.10	4	1.06	0.97	1.15
L_night_																	
<55	4867	55	1			21	1			9	1			4	1		
55–60	362	47	1.01	0.97	1.04	17	1.02	0.97	1.06	7	1.02	0.97	1.07	4	1.01	0.93	1.09
>60	130	40	1.01	0.95	1.07	13	0.97	0.89	1.04	5	0.91	0.83	1.00	3	1.13	0.98	1.30

LSOA, lower layer super output area.

^a^Adjusted for age, sex, area-level deprivation, ethnicity, smoking, and PM_2.5_.

^b^Mean number of deaths per LSOA.

In linear models, there was a small non-significant association between daytime noise and stroke mortality in adults (RR 1.02, 95% CI 0.98–1.06). Among the elderly linear models provided non-significant evidence for positive associations for the noise metrics with all cardiovascular and stroke mortality (Supplementary material online, *Table S5*). Interactions for day- and nighttime noise with area-level deprivation were non-significant in all mortality models.

## Discussion

In one of the Europe's largest cities, we found that long-term exposure to daytime road traffic noise was associated with small increased risks of cardiovascular disease and all-cause mortality in the general population. Strongest associations were observed between daytime road traffic noise and hospital admissions for stroke, particularly in the elderly (≥75 years). In general, evidence for the harmful health effects of nighttime road noise was weaker. We found no evidence for differences in noise-health associations by area-level deprivation.

Few studies have examined associations between road traffic noise and the health outcomes studied here. We found no studies for all-cause mortality and most studies available were for ischaemic heart disease.^6,30^ A meta-analysis reported an overall RR (1.08 per 10 dB L_dn_) for IHD (prevalence, incidence, and mortality) at noise levels ranging from <50 to >75 dB when adjusting at minimum for age and sex.^6^ A recent cross-sectional study not included in the meta-analysis reported that traffic noise was associated with 1.72-fold increased risk of self-reported prevalence of IHD in India.^30^ Our study found somewhat inconsistent associations for IHD and all cardiovascular disease mortality, similar to the findings of a cohort study from the Netherlands where a positive non-significant association was observed between road noise L_den_ >65 vs. ≤50 dB(A) and all cardiovascular mortality after adjustment for air pollution, but no associations were seen with IHD.^18^

Our study provides evidence for increased risk of hospital admissions for stroke in the elderly and was suggestive of associations with stroke mortality in the elderly, for which few comparable studies are available. Analysis in a Danish cohort^15,16^ reported significant associations between noise >60 dB and increased risk of stroke admissions among people over 64.5 years,^15^ and also with incident ischaemic strokes^16^ after adjusting for air pollution, but no association with fatal ischaemic stroke.^16^ However, no association was observed between road traffic noise and cerebrovascular mortality in a Dutch cohort.^18^ Two further studies combining stroke and heart disease outcomes did not find significant associations. A small multi-national cross-sectional study reported positive association between road noise and self-reported ‘heart disease and stroke’ in those ≥65 years old, and for stroke alone (though non-significant), but sub-sample analyses suggested confounding by air pollution exposure.^21^ In the Dutch GLOBE study cohort no association between road traffic noise and hospital admission was observed for the combined ‘IHD or cerebrovascular disease’ after adjusting for particulate pollution, or restriction to those ≥65 years.^20^

Stronger associations for stroke compared with other cardiovascular diseases is biologically plausible in light of the consistent evidence linking noise exposure to hypertension,^11^ a leading cause of stroke.^31^ Stronger associations with stroke than all cardiovascular disease or IHD were also seen in our study on aircraft noise.^25^ In the current study, noise–health relationships were stronger among the elderly when compared with all adults, which suggests that older people may be more vulnerable to the effects of road traffic noise. However, it is also possible that those aged ≥75 years have lived in the same address for a longer time period or spend more time at home, and therefore have less misclassification in their exposure estimates.

There are several limitations to this study. The exposure model used is likely to over-estimate noise at low exposure levels and underestimate noise in areas with heavily trafficked minor roads. This might have impaired detection of dose–response relationships in linear analyses, especially for nighttime noise (when exposure levels are lower), and was part of the rationale for using exposure categories which may reduce resultant misclassification. Averaging noise estimates at the small-area level decreased the spatial variation in exposure and likely reduced our power to detect any true positive associations. The exposure models did not take into account population activities (e.g. working and commuting outside residential areas) or residence characteristics (e.g. windows towards road vs. inner yard, building materials) and we did not have data on residential histories which may have introduced further exposure misclassification, resulting in bias towards the null. Currently, there are no models that account for time activity patterns of the population, however; misclassification due to activities is likely to affect less night- than daytime exposure. As our results for day and night were reasonably consistent, misclassification cannot fully explain our findings. The number of areas affected by rail or aircraft noise in the study area was limited and therefore their individual effects could not be examined with the spatial method used. Rail and aircraft noise have different acoustic characteristics and are not simply additive with road noise.^7^ However, rail and aircraft noise did not correlate with road traffic noise across the study area suggesting that they would not confound the observed associations.

Because of the ecological study design, it is possible that the population-level associations we observed are not representative of those at individual level. In addition, residual confounding is likely to affect the results as we were not able to adjust for individual-level confounders such as socioeconomic status, health behaviours, and co-morbidities. The number of events in each small area was low, but the statistical model was able to deal with the scarcity of the data through the hierarchical/spatially structured effects.

The main strength of this study is that we explored the health effects of road traffic noise across the total population of a metropolitan area including all registered deaths and cardiovascular hospital admissions. We used a sophisticated noise model which provides detailed spatial variation in noise levels allowing for the epidemiological analysis of relatively small health effects. We also adjusted for the main possible confounders including air pollution; however, replication of our findings in large individual-level studies with more detailed information on exposure modifiers and confounders is needed.

In conclusion, this is the largest study to date to investigate environmental noise and cardiovascular disease in the general population. Results suggested small increased population risks of all-cause mortality and cardiovascular mortality and morbidity, particularly of stroke in the elderly, at moderate levels of road noise exposure. Findings are consistent with the larger body of evidence linking traffic noise exposure with hypertension.

## Supplementary material

Supplementary material is available at *European Heart Journal* online.

## Funding

This work was supported by the UK Natural Environment Research Council, Medical Research Council, Economic and Social Research Council, Department of Environment, Food and Rural Affairs, and Department of Health (NE/I007806/1, NE/I00789X/1, NE/I008039/1) through the cross-research council Environmental Exposures & Health Initiative. The work of the UK Small Area Health Statistics Unit (SAHSU) is funded by Public Health England as part of the MRC-PHE Centre for Environment and Health, and by the UK Medical Research Council. Funding to pay the Open Access publication charges for this article was provided by the UK Natural Environment Research Council, Medical Research Council, Economic and Social Research Council, Department of Environment, Food and Rural Affairs, and Department of Health (NE/I007806/1, NE/I00789X/1, NE/I008039/1) through the cross-research council Environmental Exposures & Health Initiative.

**Conflict of interest:** none declared.
